# How do we incorporate patient views into the design of healthcare services for older people: a discussion paper

**DOI:** 10.1186/s12903-018-0513-7

**Published:** 2018-04-06

**Authors:** Paul R. Brocklehurst, Gerald McKenna, Martin Schimmel, Anastassia Kossioni, Katarina Jerković-Ćosić, Martina Hayes, Cristiane da Mata, Frauke Müller

**Affiliations:** 1 Health Services Research, Bangor University, Gwynedd, Wales; 2 Restorative Dentistry and Prosthodontics, Queen’s University, Belfast, Northern Ireland; 3 Gerodontology, University of Bern, Bern, Switzerland; 4 Gerodontology, National and Kapodistrian University of Athens, Athens, Greece; 5 Innovations in Preventive Care, University of Applied Sciences Utrecht, Utrecht, The Netherlands; 6 Restorative Dentistry, Cork Dental School, Cork, Ireland; 7 Gerodontology and Removable Prosthodontics, University of Geneva, Geneva, Switzerland

**Keywords:** Older people, Healthcare service design, Oral health, Co-production and co-creation

## Abstract

**Background:**

Across the European Union costs for the treatment of oral disease is expected to rise to €93 Billion by 2020 and be higher than those for stroke and dementia combined. A significant proportion of these costs will relate to the provision of care for older people. Dental caries severity and experience is now a major public health issue in older people and periodontal disease disproportionately affects older adults. Poor oral health impacts on older people’s quality of life, their self-esteem, general health and diet. Oral health care service provision for older people is often unavailable or poor, as is the standard of knowledge amongst formal and informal carers. The aim of this discussion paper is to explore some of the approaches that could be taken to improve the level of co-production in the design of healthcare services for older people*.*

**Main text:**

People’s emotional and practical response to challenges in health and well-being and the responsiveness of systems to their needs is crucial to improve the quality of service provision. This is a particularly important aspect of care for older people as felt, expressed and normative needs may be fundamentally different and vary as they become increasingly dependent. Co-production shifts the design process away from the traditional ‘top-down’ medical model, where needs assessments are undertaken by someone external to a community and strategies are devised that encourage these communities to become passive recipients of services. Instead, an inductive paradigm of partnership working and shared leadership is actively encouraged to set priorities and ultimately helps improve the translational gap between research, health policy and health-service provision.

**Discussion:**

The four methodological approaches discussed in this paper (Priority Setting Partnerships, Discrete Choice Experiments, Core Outcome Sets and Experience Based Co-Design) represent an approach that seeks to better engage with older people and ensure an inductive, co-produced process to the research and design of healthcare services of the future. These methods facilitate partnerships between researchers, healthcare professionals and patients to produce more responsive and appropriate public services for older people.

## Background

Compared to two decades ago, many older people in the United Kingdom will have most or all of their natural teeth [1]. Costs for the treatment of oral disease is expected to rise to €93 Billion by 2020 across the European Union, higher than those for stroke and dementia combined [2]. A significant proportion will relate to the provision of care for older people [3]. Many in this population were not exposed to fluoride in their childhood and nutritional advice was scarce. As a result, caries severity and experience are now a major public health issue in older people [4, 5]. Gum disease disproportionately affects older adults and when dental implants are present, peri-implantitis may lead to implant failure [6]. Self-care deteriorates with increasing age, dry mouth prevalence increases due to poly-pharmacy and diets become rich in sugars, further increasing the risk of future disease. Overall, poor oral health impacts on older people’s quality of life, their self-esteem, general health and diet [7–10]. Oral health care service provision for older people is often unavailable or poor, as is the standard of oral health literacy amongst formal and informal carers [11–13]. Equally, the provision of care is not homogeneous and is delivered by many different types of healthcare worker [11–13]. Access to domiciliary services is difficult and admission to hospital for dental problems is distressing and costly [14, 15]. Income-related inequality in dental service utilisation and oral health inequalities amongst older people is common [16]. As older peoples’ independence deteriorates, all these factors are compounded further.

The World Health Organisations report on healthy aging calls for systems of care that are fit-for-purpose and evidence based [17]. Birch argues that there are four components for a health-needs based approach to planning care: population size [P], felt and expressed need in this population [H/P], the type and level of services required to meet these needs [Q/H] and the efficiency of the healthcare sector to meet these needs [18]. The aim of this discussion paper is to explore some of the approaches that could be taken to improve the level of co-production in the research and design of healthcare services for older people, thereby providing an understanding of the type and level of services required to meet their expressed need.

### Importance of co-production in older people research

People’s emotional and practical response to challenges in health and well-being and the responsiveness of systems to their needs is crucial to improve the quality of service provision [19]. This is a particularly important aspect of care for older people as felt, expressed and normative needs may be fundamentally different and vary as they become increasingly dependent [20]. Patient and public involvement is playing an increasingly important role in health and social care research and the design of service provision [21]. Engagement is key and helps address the challenges related to translation and implementation in complex organisational settings [22]. Again, this is a key consideration in gerodontology, given the range of contexts of care.

Co-production shifts the design process away from the traditional ‘top-down’ medical model, where needs assessments are undertaken by someone external to a community and strategies are devised that encourage these communities to become passive recipients of services [23]. Instead, an inductive paradigm of partnership working and shared leadership is actively encouraged to set priorities and ultimately help improve the translational gap between research, health policy and real world practice [24–26]. To ensure an inductive process underlies the design of healthcare services for older people (both dependent and independent), a number of methodological approaches could be undertaken and the most commonly used here are:

1. Priority Setting Partnerships
2. Discrete Choice Experiments
3. Core Outcome Sets
4. Experience-Based Co-Design

### Priority setting partnerships

Priority Setting Partnerships (PSPs) incorporate users’ perspectives to help prioritise health and social care as well as research agendas and ensure they are patient-centred [27, 28]. PSPs were developed by the James Lind Alliance to help mitigate the asymmetrical relationships that often exist between researchers and users of healthcare services. They are based on a consensus methodology and use a modified Nominal Group Technique to produce a series of sequential steps to build consensus. This structured approach ensures the narratives of users of services are heard and helps counter the ‘top-down’ medical model that can dominate healthcare services [29–31].

Two pilot PSPs have already been undertaken in the United Kingdom (UK) and The Netherlands [13, 32]. Key stakeholders were asked to explore a series of stem questions for discussion and present their views. A shared ranking exercise was then undertaken after further structured small group discussions. For these studies, preliminary meetings were held with the following stakeholder groups:

1. Users of services who were older people;
2. Carers of older people;
3. Third sector e.g. older people charities;
4. Specialists e.g. geriatricians, gerodontologists, care-home managers and dental public health consultants.

Based on the Nominal Group Technique, each group took part in a facilitated discussion to identify key local priorities for health and well-being, how health and social care services could be best organised to address current and future needs and where the future priorities for service provision and research in health and social care should lie. Each group was facilitated by one of the research team and started by exploring the following stem questions:

1. What aspects of oral health are important for you now?
2. What aspects of oral health would be important to you as you lose your independence?
3. How should we best prevent dental disease in older people?
4. What does good dental care look like (as older people become increasingly dependent)?
5. What would you fear happening to your mouth that is, what negative outcomes would you want to avoid as you lose your independence?
6. What are the important research questions to answer?

The detailed methods are described by Brocklehurst et al. [13]. Following the first stage of PSP meetings, two or three members of each group were then asked to participate in a final meeting to review the collated information. This meeting was facilitated and led by a member of the ‘user’ group to ensure that the results of the PSP were grounded in service-user perspectives. The views of each preceding group were highlighted question by question, discussed, refined and then placed into a list of priorities.

The key priorities that emerged from these pilots were:

1. Identify key issues for older people from their perspective;
2. Assess the perceived oral health needs of the aging population to determine the scope and size of the problem;
3. Incorporate patient’s perspectives into the ‘best practice’ in the prevention and treatment of oral diseases for older people;
4. Identify the training needs for the dental profession arising from 3;
5. Increase awareness of the importance of good oral health among older people, caregivers and healthcare professionals;

#### Discrete choice experiment

Discrete choice experiments (DCEs) elicit respondents’ preferences and measure trade-offs between different levels of attributes for dental service provision. In addition to DCEs, best-worst scaling can be utilised to choose the best and worst level of a given attribute, which reduces the level of cognitive burden on participants. DCEs have been found to be particularly useful in establishing prioritisation frameworks and are based on two fundamental assumptions [33–35]. Firstly, that healthcare interventions and services can be described by a set of attributes. Secondly, that these attributes can be valued by an individual [34].

DCE methods combine random utility theory, consumer theory, experimental design theory and econometric analysis [36]. The strength of a DCE approach is that it can quantify respondents’ trade-off preferences between different levels of attributes, by obliging participants to choose between them. This enables researchers to estimate the probability that a person chooses a particular level on an attribute, relative to defined alternative choices. These probabilities are calculated with the assumption that the actual choices participants make are based on a well ordered set of preferences. As a result, the method maps well onto the judgment ecology involved in commissioning decisions, where decisions to invest in one service or another have an opportunity cost i.e. can’t be invested elsewhere. Best-Worst Scaling (BWS) is a form of DCE that reduces the cognitive burden on participants and so is particularly suited for older people, where some degree of cognitive impairment may be present [37]. In a BWS study, participants are asked to choose the best and worst (or most and least) level of a given attribute.

A pilot DCE is currently underway in the UK and Ireland. Based on the results of the PSP described above, principal attributes and their corresponding levels of these attributes were chosen (Table 1). The DCE was developed with a Patient and Public Involvement group of older people, based in a Foundation NHS Trust in England.

**Table 1:** Attributes and levels chosen for the pilot DCE

Attributes	Levels
Type of healthcare professional	1. Dental surgeon 2. Another trained member of the dental team
Type of activity undertaken	1. Examination (“check-up”) 2. Treatment
Where activity is undertaken	1. At home 2. In a dental practice 5 miles away 3. In a dental practice 10 miles away 4. In a dental practice 15 miles away

### Core outcome sets

The selection of appropriate outcomes is crucial when designing clinical trials to directly compare the effects of different health service models in ways that minimize bias. Systematic reviews of clinical trials are commonly used to form policy guidelines. However, there is a growing recognition that insufficient attention has been paid to the level of consistency in the use of outcomes measure and the impact this has on the heterogeneity of included studies and the ability to undertake meta-analyses [38–41]. In a number of clinical disciplines, these issues are being addressed through the development and use of an agreed standardized collection of outcomes, known as a Core Outcome Set (COS), which are then measured and reported in all trials [42]. One critical element of COS design is that they should include the views of patients [40]. COS studies that have adopted this approach have identified outcomes that have not been previously identified, highlighting the importance of this principle [43, 44].

The development of COS commonly starts with a systematic review. To this end, an Effective Practice and Organisation of Care Cochrane review entitled “Strategies to prevent oral disease in dependent older people” is currently on-going and a COS for older people has been registered with COMET [45]. Consensus methods are then used to understand ‘what’ to measure, followed by ‘how’ and ‘when’. To facilitate this, the COSMIN (COnsensus-based Standards for the selection of health Measurement INstruments) checklist can be used as a tool for developing studies of the validity and reliability of the proposed measurement instruments [46]. COSMIN describes the necessary design requirements for the assessment of those measurement properties. In addition, the feasibility of measurement is another important consideration [47].

Consensus methods include expert panel meetings, Delphi surveys, Nominal Group Techniques, focus groups, individual interviews and individual questionnaires [32, 48–52]. Given the similarity of some of these approaches to those outlined for the PSP, these could be undertaken simultaneously. Anonymous and electronic voting methods haven proven helpful (on-site and remotely) at the final consensus stage [40]. Stakeholders are asked to score each outcome from a long list of identified outcome measures gleaned from the systematic review and the previous stages of the process. Subsequent approaches for the final selection of the COS include the scale proposed by GRADE: 1 to 3 signifies an outcome of limited importance, 4 to 6 important but not critical, and 7 to 9 critical [40]. A number of rounds can then be held in which responses are summarised and fed back to the stakeholder groups producing a refined version. Consensus regarding whether an outcome should be included in the COS can then be defined as 70% or more of the respondents scoring the measure between 7 to 9 and fewer than 15% scoring it as 1 to 3. Equally, consensus that an outcome is not included in the COS can be defined as 70% or more scoring it as 1 to 3 and fewer than 15% scoring it as 7 to 9. All other score distributions then indicate lack of agreement for inclusion.

### Experience-based co-design

Engagement is key and helps address the challenges related to translation and implementation in complex organisational settings [53]. Experience based co-design (EBCD) is a participatory action research approach that puts users at the centre of the design process. It draws on narrative interviews with patients about their experiences of care, as well as staff interviews and ethnographic observations [54]. By identifying and understanding how patients’ subjective experiences are shaped as they engage with the health service, it is possible to better design these experiences rather than simply re-design processes of care [55]. This shifts the design process from the traditional “top-down” medical model to an inductive paradigm of partnership working and shared leadership with patients [56, 57].

Careful observation, measurement, recording, analysis and interpretation of patients’ subjective experiences are essential to appreciating what is working well in healthcare, what needs to change, and how to go about making improvements [58]. Patients first immerse and record their daily experiences using a range of self-documentation exercises (scrapbooks or storytelling exercises). They then are encouraged to articulate their feelings about their lived experiences using images and collages. Patients are then asked what an “ideal” experience would look like, encouraging them to think about how the experience should feel in abstract terms. This is facilitated using collages and maps of processes. Following this, participants are asked to imagine how they want to feel and are encouraged to create solutions that will provide their aspired experiences.

Patient interviews are video recorded and analysed for “touch-points”, key moments of interaction between patients, carers and care systems where quality can be improved [55]. A “trigger film” illustrating this analysis is shown to both patients and healthcare professionals, who then work together to implement agreed improvements. Local interviews have traditionally been used as the basis for EBCD, but recent research has shown that nationally collected video interviews can also be used effectively [59].

## Conclusion

The four approaches highlighted above represent some of the more common methods of ensuring an inductive and co-produced approach to the research and design of healthcare services. These are summarised in Table 2. All place an emphasis on building consensus with stakeholders, in order to ensure the research process or design of the service is centred around the expressed needs of the target population. With respect to older people, it is important to emphasise that those over sixty-five years of age are not one homogenous group and so further differentiation is important [60], particularly in respect of chronological/physiological ageing, dependence/independence and their home setting (e.g. living in a home versus living at home).

**Table 2:** Summary of the main elements of each inductive approach

Approach	Design	Outcome
Priority Setting Partnership	An inductive and partnership approach using focus groups to build consensus	Identify key issues and priorities for end-users and where the research evidence requires strengthening
Discrete Choice Experiment	Presents choice sets to end-users to force decisions about the most preferred combination of attributes and values	Hierarchy of preferred options for the design of healthcare services
Core Outcome Set	Iterative and inductive approach using a broad range of stakeholders to determine the most important outcomes for a patient group	Consensus on the key primary outcome measures to be collected for experimental research designs
Experience-based Co-Design	Collates audio and visual evidence and uses an iterative design process to incorporate the ‘felt’ views of the end-user	Uses the emotional experience of the end-user to better design care-pathways and provision of healthcare

As highlighted in this paper, co-production has been described as a key principle of healthcare and governments have increasingly called for more explicit attention to facilitate partnerships between professionals and beneficiaries in co-producing public services [61, 62]. Equally, an increasing number of funding bodies see Patient and Public Involvement as key and recognise the need for researchers to account for the views of population that they are studying. Examples of co-production in healthcare provision include: (1) co-commissioning of services; (2) co-design of services; (3) co-delivery of services and (4) co-assessment [63, 64]. However, there remain challenges in the implementation of a co-produced approach. It can remain difficult to move beyond ‘researcher-centric and ‘professional-centric’ priorities, boundaries and culture, with researchers and professionals failing to account for the end-user [65, 66].

From a research perspective, if a number of countries across Europe were to undertake a PSP and DCE, this could have real value for driving policy decisions forward at a country-wide and European Union level. Developing a COS would enable researchers to be consistent in the outcome measures chosen in experimental evaluative designs, thereby increasing the power of subsequent meta-analyses in secondary research. Likewise, EBCD offers a method of locally tailoring services to address the specific needs of older people.

In the healthcare sector, given that “public services face an unprecedented set of challenges: increasing demand, rising expectations, seemingly intractable social problems and, in many cases, reduced budgets”, “radical innovation in public services now needs to move from the margins to the mainstream” [67].

## Abbreviations

BWS: Best-Worst Scaling
COS: Core Outcome Set
DCE: Discrete choice experiments
PSPs: Priority Setting Partnerships
RRR: Rapid Realist Review
